# Unraveling the complex interplay: self-esteem, impostor phenomenon, proactive personality, and their influence on career satisfaction

**DOI:** 10.3389/fpsyg.2025.1583454

**Published:** 2025-04-28

**Authors:** Erkan Taşkıran, Gülşah Gençer Çelik, Nihal Kartaltepe Behram, Esra Dinç Elmalı, Gökten Öngel

**Affiliations:** ^1^Department of Tourism Administration, Akçakoca School of Tourism Administration and Hotel Management, Düzce University, Düzce, Türkiye; ^2^Department of Business Management, Vocational School, İstanbul Beykent University, Istanbul, Türkiye; ^3^Department of Management and Organization, Faculty of Business Administration, Marmara University, Istanbul, Türkiye; ^4^Child Health and Diseases Department, Istanbul Training and Research Hospital, Istanbul, Türkiye

**Keywords:** self-esteem, impostor phenomenon, proactive personality, career satisfaction, employees

## Abstract

**Background:**

Based on Social Identity Theory (SIT) and Self-Determination Theory (SDT), this study proposed a moderated mediation model in which the impostor phenomenon was established as an intervening instrument that highlighted why employees were more likely to be satisfied with their careers under the existence of self-esteem, and the indirect effect of self-esteem on career satisfaction via the impostor phenomenon was conditional on proactive personality.

**Methods:**

The data required to fulfill the study’s purpose were collected through the survey technique. Employees (*N* = 376) working in customer relations, branch banking support, commercial transactions, etc., units at the head office of a well-known private bank operating in Istanbul, Turkey, were surveyed.

**Results:**

The findings obtained from the study revealed that self-esteem has a positive effect on career satisfaction and a negative impact on the impostor phenomenon. It was also found that the impostor phenomenon has a negative effect on career satisfaction. Moreover, it was discovered that the impostor phenomenon has not mediated the relationship between self-esteem and career satisfaction. However, a proactive personality has moderated the effect of the impostor phenomenon on career satisfaction. Furthermore, a proactive personality moderated the indirect effect of the impostor phenomenon on the relationship between self-esteem and career satisfaction.

**Conclusion:**

This study underscores a novel intersection of psychological constructs -self-esteem, the impostor phenomenon, and proactive personality- and their profound implications for career satisfaction, bringing us one step closer to understanding the complex dynamics of employee satisfaction within the professional realm.

## 1 Introduction

The impostor phenomenon, which Clance and Imes (1978) introduced to the study of organizational behavior, is characterized as a personal feeling of being an intellectual fraud. It is a motivational disposition in which people who have achieved a certain level of success have a sense of being a fraud (Ross and Krukowski, 2003). Individuals with the impostor phenomenon feel unworthy of their achievements and have an unavoidable fear of someday being discovered as the fraud they believe themselves to be (Schubert, 2013). Imposters need a sense of internalization of their success (Yaffe, 2020). Individuals experiencing impostorism have difficulty attribution of their performance to their actual competence. They attribute their successes to external factors such as luck, unstable factors, or the help they receive from others, and they attribute their setbacks to their professional inadequacies (Kaur and Jain, 2022).

Feelings of having an impostor phenomenon have been linked to poor psychological functioning, including low self-esteem (Peteet et al., 2015). The available literature has found a negative relationship between self-esteem and the impostor phenomenon. Previous research (Sonnak and Towell, 2001; Lige et al., 2017; Neureiter and Traut-Mattausch, 2016; Schubert and Bowker, 2019; Chrisman et al., 1995; Cozzarelli and Major, 1990; Topping and Kimmel, 1985) revealed this negative relationship between self-esteem and impostor phenomenon. In this context, the role of self-esteem in the impostor phenomenon should not be underestimated (Schubert, 2013). On the other hand, as has been suggested in many studies related to career research (Joo and Park, 2010), career success and career satisfaction are used interchangeably in this research. Self-esteem, as a household word (Baumeister et al., 2003), can impact an individual’s performance to a large extent (Akgunduz, 2015). A high level of self-esteem is helpful to individuals in many contexts, such as career-related issues (Sabelnikova, 2021). Greenhaus (1971) suggested that individuals with high self-esteem were more concerned with career satisfaction. Atac et al. (2017) proposed that individuals with high self-esteem tend to take responsibility and feel more control over their careers. Furthermore, no research was available on the adverse or non-linear effect of high self-esteem on career satisfaction (Sabelnikova, 2021). Joo and Ready (2012) stated that the subjective part of career success refers to job or career satisfaction. Within this context, job satisfaction can be considered within the scope of career satisfaction. The majority of existing studies in the literature to date (Sabelnikova, 2021; Cai et al., 2015; Mocheche et al., 2017; Zafar et al., 2014; Kuster et al., 2013; Ahmed, 2012; Kammeyer-Mueller et al., 2008; Alavi and Askaripur, 2003) have examined the relationship between individuals’ self-esteem and career satisfaction including career success and job satisfaction. Accordingly, although studies on self-esteem, the impostor phenomenon and career satisfaction exist in the available literature, no studies focused on the indirect effect of variables such as the impostor phenomenon.

There is a growing body of literature on the impostor phenomenon and its role in various career outcomes. Still, more needs to be known about the mediating or moderating personality variables that affect impostorism (Cokley et al., 2018). Jawahar and Liu (2016) suggested that in today’s dynamic work environment, a proactive personality is needed as a highly relevant personality. According to Bateman and Crant (1993), individuals with a proactive personality can affect their environment by scanning for opportunities, showing initiative, taking action and persevering until they reach closure by bringing about change. Individuals with proactive personalities may feel supported. They may affect the association between the impostor phenomenon and career satisfaction. Although many studies, as discussed above, have examined the research variables, there is a lack of research on concerning self-esteem, impostor phenomenon, proactive personality and career satisfaction together. At this point, there seems to be a need to investigate the research gap that reveals such an association’s existence. Therefore, the main focus of this research is whether the indirect effect of self-esteem on career satisfaction through the impostor phenomenon is conditional on proactive personality. In addition to examining the main effect of self-esteem on career satisfaction and the impostor phenomenon, this study targeted to explain the direct and indirect impact of proactive personality in these associations. Firstly, a proactive personality was assumed as a potential moderator of the relationship between the impostor phenomenon and career satisfaction. Secondly, the mediator effect of the impostor phenomenon in the relationship between self-esteem and career satisfaction might differ with a proactive personality. The career satisfaction of an individual experiencing the impostor phenomenon may decrease, but having a proactive personality varies this relationship. Career satisfaction may increase due to a proactive personality structure even if the individual experiences an impostor.

In this setting, drawing upon the social identity theory (SIT) and self-determination theory (SDT), the current research seeks to clarify how the impostor phenomenon mediates the connection between self-esteem and job satisfaction. Additionally, a moderated mediation model was established to examine the moderating role of proactive personality on the indirect effect of self-esteem on career satisfaction through the impostor phenomenon.

The contribution of this study can be explained as follows. Firstly, a detailed literature review revealed little research investigating the abovementioned variables based on the model proposed within this study. Thus, the present study is expected to contribute to the relevant literature. Secondly, this study aimed to provide a rationale for whether the impostor phenomenon affects individuals’ career satisfaction who have remarkable career advancements and whether proactive personality, as an individually assessed variable, alters this relation. By seeking answers to these questions, it is expected to comprehend the role of self-esteem and proactive personality in the career satisfaction of employees experiencing the impostor phenomenon.

The structure of the study consists of a review of available literature on self-esteem, career satisfaction, the impostor phenomenon and proactive personality, along with the theoretical framework drawn from the perspective of social identity theory and self-determination theory, explaining the association between constructs, the mediating effect of the impostor phenomenon, the moderating effect of proactive personality and the moderated mediation model, respectively. Afterward, after analyzing the hypotheses developed within the scope of the study, the paper is concluded with the presentation of the research findings, discussion, implications, limitations and recommendations for future research.

## 2 Theoretical framework and development of hypotheses

### 2.1 Theoretical framework

#### 2.1.1 Self-esteem

Self-esteem, which plays a moderating role socially and psychologically, refers to a subjective evaluation that can be defined by how important and valuable an individual considers themself (Feng et al., 2025). Alimbekov et al. (2025) define self-esteem as the involvement of self-evaluation, the cultivation of abilities, interaction with the environment, and a holistic assessment. They encompass crucial characteristics such as self-confidence, self-respect, and satisfaction with one’s identity. When individuals with low and high self-esteem are compared, they tend to evaluate themselves more positively than those with low self-esteem. They may experience stronger feelings regarding self-worth (Brown and Marshall, 2006). In addition, individuals with high self-esteem can better protect themselves against psychological problems such as anxiety and depression (Bravata et al., 2019). Self-esteem can be explained precisely by how much individuals value themselves. The individual’s self-knowledge, recognition and self-worth are considered within self-esteem. Therefore, self-esteem is a perception rather than a reality (Baumeister et al., 2003).

#### 2.1.2 Impostor phenomenon

The impostor phenomenon is defined as having feelings of fraudulence because the person does not attribute their accomplishments to their competencies even when the contrary is present (Parkman, 2016). Research psychologists Pauline Clance and Suzanne Imes identified the impostor phenomenon after studying over 150 women in higher education who have made many significant achievements in their fields. They noticed a common theme of women attributing their achievements to external factors such as luck, timing, or other people’s help rather than internal factors such as their skills and efforts. This misattribution of success caused the participants to describe their inadequacy and fears of being “found out” as phonies despite legitimate and tangible achievements. Clance and Imes initially suggested that the impostor phenomenon only affected women. However, several current studies have indicated that feeling like an impostor affects a wide range of people in different groups, such as working professionals (Tewfik, 2022; Schow, 2021; Neureiter and Traut-Mattausch, 2016); higher education (Manongsong and Ghosh, 2021; Cisco, 2020; Hutchins and Rainbolt, 2017; Parkman, 2016; Dancy and Jean-Marie, 2014); entrepreneurs (Ladge et al., 2019); politicians (Aguiar and Redlin, 2014); managers (Downing et al., 2020; Fried-Buchalter, 1997); medical students and practitioners (Neufeld et al., 2022; Mattie et al., 2008; Oriel et al., 2004); military personnel (Stein et al., 2020) and, librarians (Barr-Walker et al., 2019).

#### 2.1.3 Career satisfaction

Career satisfaction, which involves the subjective evaluation of the work environment (Park, 2018), is an important indicator of subjective career success that captures employees’ cognitive and emotional assessment of their career-related outcomes (McKenna et al., 2016). Career satisfaction is a broad concept that refers to employees’ perceptions of both intrinsic and extrinsic aspects of their career development, including income, promotion, progress toward career goals, development opportunities and perceived career success (Baidoun and Anderson, 2024). Individuals with high career satisfaction are expected to perform better in their jobs, work more efficiently and effectively, and continue pursuing their career goals (Aburumman and Wasfi Alrweis, 2024). Thus, achieving career goals, measuring career success and expecting future development are often associated with career satisfaction (Zhu et al., 2022). In addition, career satisfaction results when individuals meet their career needs by focusing on achieving their career goals. Therefore, career satisfaction, as a result of an individual motivation process, includes tools such as organizational support and career development (Jung and Takeuchi, 2017).

#### 2.1.4 Proactive personality

Proactive personality refers to a stable tendency to take personal initiative and a desire to make a difference (Kim and Oh, 2024). The main characteristic of proactive behavior is that individuals can directly and intentionally change their current social or non-social circumstances (Bateman and Crant, 1993). Being proactive means changing things for the better in an intended direction. Proactive behavior separates individuals from the pack and organizations from the rest of the market (Bateman and Crant, 1999). Highly proactive individuals tend to take initiative, anticipate and create change, and seek opportunities rather than waiting for them to arise (Crant, 2000). Hence, proactive individuals in an organization can benefit from faster advancement, better job opportunities and career paths (Yang et al., 2011).

### 2.2 Development of hypotheses

#### 2.2.1 Self-esteem and career satisfaction

Self-esteem is an individual’s general self-evaluation of his/her competencies (Rosenberg, 1965). According to another definition, self-esteem is a personal evaluation reflecting what people think about themselves (Pierce and Gardner, 2004). On the other hand, Greenhaus et al. (2010) define career as the pattern of work-related experiences that span a person’s life. Like much career research, this study used career satisfaction and success interchangeably (Joo and Park, 2010). Therefore, career success is the positive psychological or work-related outcomes or achievements accumulated from work experiences (Judge et al., 1995). Judge et al. (1995) consider career success in two dimensions, objective and subjective and define subjective career success as including current job satisfaction, just as a career includes a current job. According to them, career satisfaction is the satisfaction individuals derive from their careers’ intrinsic and extrinsic aspects, including pay, advancement and developmental opportunities.

In the literature, there are several studies investigating the relationship between self-esteem and career satisfaction, including career success and job satisfaction (Sabelnikova, 2021; Cai et al., 2015; Mocheche et al., 2017; Zafar et al., 2014; Ahmed, 2012; Kammeyer-Mueller et al., 2008; Alavi and Askaripur, 2003; Garske, 1996; Westaway et al., 1996). Sabelnikova (2021) found a clear relationship between self-esteem and career success. It has been suggested that a significant relationship exists between self-esteem and job satisfaction (Mocheche et al., 2017). Ahmed (2012), in a study conducted on private university teachers, concluded that self-esteem and optimism significantly affect job satisfaction. Garske (1996) found a significant relationship between self-esteem and career satisfaction. Alavi and Askaripur (2003), in their study, concluded that there are significant relationships between self-esteem and the degree of satisfaction with the type and quality of work, manager or supervisor, coworkers, promotion, salary and wages, and self-esteem. Within this framework, the first hypothesis of the research is composed as given below:

**Hypothesis 1 (H1)**: Self-esteem is positively related to career satisfaction.

#### 2.2.2 Impostor phenomenon as a mediator

Issues with self-esteem constitute the basis of the impostor phenomenon. In the literature, many empirical studies examined the relationship between the impostor phenomenon and self-esteem level and revealed a negative relationship between self-esteem and the impostor phenomenon (Sonnak and Towell, 2001; Neureiter and Traut-Mattausch, 2016; Schubert and Bowker, 2019; Chrisman et al., 1995; Cozzarelli and Major, 1990; Topping and Kimmel, 1985). The relationship between the impostor phenomenon, self-worth, and self-reported and statistical instability in self-esteem was explored in a study by Schubert and Bowker (2019). The findings revealed a negative correlation between the impostor phenomenon and levels of self-esteem and a positive correlation with both self-perceived and statistical volatility of self-esteem. Neureiter and Traut-Mattausch (2016) found that diminished self-worth is a contributing factor to feelings of impostor phenomenon. Research conducted on university faculty members by Topping and Kimmel (1985) showed a significant negative association between self-worth and the impostor phenomenon. Similarly, Mascarenhas et al. (2019) concluded that there was a notable negative correlation between self-esteem and the impostor phenomenon in a study involving medical interns. Lige et al. (2017) explored the interplay among racial identity, self-esteem, and the impostor phenomenon, finding that racial identity positively influences self-esteem and negatively impacts the impostor phenomenon. Interestingly, they also found that self-esteem negatively influenced the impostor phenomenon. Considering these findings, the following hypothesis was formulated:

**Hypothesis 2 (H2)**: Self-esteem is negatively related to impostor phenomenon.

The impostor phenomenon is considered an important psychological construct regarding career development. Individuals experiencing the impostor phenomenon present themselves as talented and self-confident people. They tend to be perfectionists and fear failing by disappointing others. These individuals are also concerned about success as they think that they do not deserve the success they have achieved (Sharma, 2018). When the general characteristics of the impostor phenomenon are examined, it is clear that the careers of people suffering from it can be negatively affected. Moreover, the inability of these individuals to internalize their success and their fear that they will not achieve the same success in subsequent tasks can reduce their satisfaction with success and even make them feel worse for being successful (Clance and O’Toole, 1987).

Limited research examines the link between the impostor phenomenon and career-related issues. Those few studies suggest that the impostor phenomenon has a detrimental impact on aspects of career progression. In a study by Neureiter and Traut-Mattausch (2016), they explored the association between impostor feelings and various elements like fear of failure, success, self-esteem, career advancement, career planning, career ambition, and leadership motivation. They discovered that these impostor sentiments negatively impacted students’ career planning and efforts and undermined working professionals’ motivation to take on leadership roles. Sharma (2018) established that the impostor phenomenon adversely affects one’s career satisfaction and perceived career success. Wang et al. (2024) concluded that there is a negative relationship between impostor phenomenon and job satisfaction. Hudson and González-Gómez (2021) found a negative relationship between the impostor phenomenon and career success in their study. Considering these findings, the following third hypothesis for this study was formulated:

**Hypothesis 3 (H3)**: Impostor phenomenon is negatively related to career satisfaction.

In the literature, there is no study investigating the mediator role of the impostor phenomenon in the relationship between self-esteem and career satisfaction. Only Neureiter and Traut-Mattausch (2016) evaluated the impostor phenomenon within the scope of career development and tested a model including preconditions and consequences of the impostor phenomenon. Their study assessed the impact of fears surrounding failure and success. They diminished self-esteem on feelings of being an impostor while examining the influence of these impostor feelings on career-related outcomes (such as career planning, ambition, and leadership motivation). The research findings indicated that the fear of failure and success, along with low self-esteem, contribute to developing impostor feelings. These feelings, in turn, affect career planning, unobservable and observable career efforts, and motivation to assume leadership roles.

People suffering from the impostor phenomenon live their lives believing that their success is due to some luck or mistake. They constantly fear being found out to be less intelligent or less talented9. Research has shown that people with low self-esteem are more likely to suffer from the impostor phenomenon. In addition, since people suffering from the impostor phenomenon think that they do not deserve the success they have achieved, career success, which is considered important in terms of personal career development and career satisfaction, can be viewed as a sub-dimension and may be negatively affected. In this case, the impostor phenomenon may mediate the relationship between self-esteem and career satisfaction. The related hypothesis is given below:

**Hypothesis 4 (H4)**: Impostor phenomenon mediates the relationship between self-esteem and career satisfaction.

#### 2.2.3 Proactive personality as a moderator

In the literature, many studies examine the relationship between personality dimensions and the impostor phenomenon (Bernard et al., 2002; Chae et al., 1995; Beard, 1990; Kaur and Jain, 2022). In a study conducted on 190 college students, Bernard et al. (2002) concluded that the impostor phenomenon is associated with high neuroticism and low conscientiousness. They also concluded that low self-discipline and perceived competence, as well as depression and anxiety, are important characteristics of the impostor phenomenon. Chae et al. (1995) utilized personality inventories to measure personality dimensions in their research with 654 Korean participants to determine the relationship between impostor phenomenon and personality. As a result, they concluded that there is a relationship between the impostor phenomenon. According to this result, it was seen that people with the impostor phenomenon are more introverted types than extroverted. In addition, the study concluded that there were significant relationships between the impostor phenomenon and neuroticism and honesty from the dimensions of the NEO-PI-R personality inventory. According to this result, it was seen that people with the impostor phenomenon had high levels of neuroticism and low levels of conscientiousness.

Proactive personality is a personality type that is relatively unconstrained by situational forces and influences environmental change. Proactive people scan for opportunities, show initiative, take action, initiate change and sustain it (Bateman and Crant, 1993). Subjective career success is also related to a proactive personality. Proactive individuals are more effective in shaping their work environment. Behaving like this affects career satisfaction in two ways. Firstly, proactive individuals have a greater sense of self-determination and self-efficacy in their professional lives, which may impact career satisfaction. Secondly, individuals with proactive personality traits make more effort to choose and create work environments suitable for their professional needs and values. The harmony between the individual and the work environment can also improve career satisfaction (Seibert et al., 1999).

As can be seen, personality is a variable related to both the impostor phenomenon and career satisfaction. In this context, a proactive personality may moderate the relationship between the impostor phenomenon and career satisfaction. In other words, the negative effect of the impostor phenomenon on career satisfaction may be reduced by proactive personality traits. Within this framework, the fifth hypothesis of this study was formed as below:

**Hypothesis 5 (H5)**: Proactive personality moderates the relationship between impostor phenomenon and career satisfaction.

Proactive personality is recognized as a stable tendency to take personal initiative in various activities and situations. Proactive people are relatively unconstrained by situational forces and influence environmental change. Empirical evidence suggests that a proactive personality is a unidimensional construct positively related to a range of important individual and organizational outcomes (Seibert et al., 2001). In this context, the greater an individual’s proactive personality trait, the greater the indirect effect of the impostor phenomenon on the relationship between self-esteem and career satisfaction, and conversely, the weaker an individual’s proactive personality trait, the weaker the indirect effect of the impostor phenomenon on the relationship between self-esteem and career satisfaction. In this context, this article proposes the following hypothesis.

**Hypothesis 6 (H6)**: The indirect effect of self-esteem on career satisfaction via impostor phenomenon is conditional on proactive personality.

#### 2.2.4 Self-determination theory (SDT)

Self-determination theory, developed by psychologists Edward Deci and Richard Ryan in 1975, focuses on motivation, personality development and wellness. The concept of self-determination refers to individuals’ experience of freedom in initiating their behaviors, self-management and making confident choices by trusting themselves while making decisions. SDT assumes that autonomous or intrinsic motivation (engaging in an activity by one’s own choice) is essential in sustaining behaviors that encourage wellness and high-quality performance, whereas controlled or extrinsic motivation (i.e., engaging in an activity for contingent rewards or power dynamics) leads to decreases in motivation, wellbeing, and work performance and engagement (Ryan and Deci, 2017; Ryan and Deci, 2020). SDT can provide a useful theoretical framework for explaining self-esteem and the impostor phenomenon. According to SDT, if individuals experience ongoing satisfaction of their basic needs developmentally, they tend to become secure within themselves and experience a sense of true self-worth and self-esteem that is relatively stable and not a source of focus or concern. In contrast, if people experience deficiencies in satisfaction of their basic needs, their sense of self will be less secure. They will likely strive for extrinsic goals or standards that imply significance or worth (Moller et al., 2006). In this context, this study asserted that people with low self-esteem often feel impostor phenomenon than people with high self-esteem.

#### 2.2.5 Social identity theory (SIT)

**Figure 1:**
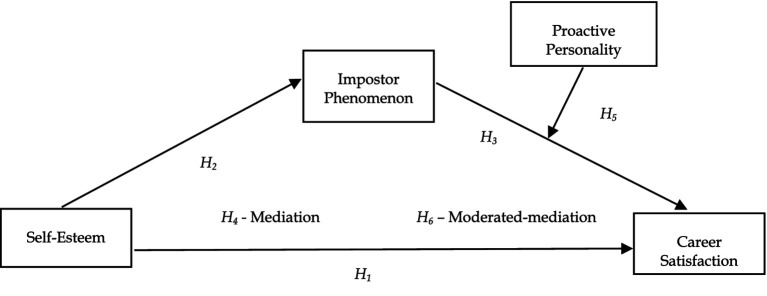
Proposed research model.

Social identity theory (SIT), as put forth by Tajfel (1978), is the idea that an individual’s self-concept stems from their affiliation with social groups. Tajfel (1972) characterized social identity as the understanding an individual has of their membership in specific social groups, coupled with the emotional significance and value they attach to being a part of those groups. Per the SIT, an individual’s perception of self varies within the group context, suggesting that impactful group involvement can supersede individual identity, replacing it with a social one (Demirtaş, 2003). Individuals suffering from the imposter phenomenon perceive themselves as included in a group despite not meeting the necessary qualifications for group membership. Regarding SIT, the impostors perceive the group they are a part of positively but do not see themselves fitting into that category. Individuals with low self-esteem attempt to meet their self-esteem needs by belonging to a group they view positively. However, as they perceive the difference between their individual qualities and those of the group, they feel like an impostor. Within the relations among variables, the model of the study is presented in Figure 1.

## 3 Method

### 3.1 Sample and procedure

The data required to fulfill the purpose of the study were collected through the survey technique, one of the quantitative research methods. The research was conducted on the employees working in customer relations, branch banking support and commercial transactions, etc., units at the head office of a well-known private bank operating in Istanbul, Turkey, which has an important place in the banking sector. The reason for choosing the banking sector for the research was the idea that to work in this sector, in addition to technical knowledge, it is necessary to establish and manage networks. After conducting interviews with the senior management of the relevant bank with the help of personal contacts, all headquarters employees, except for the senior management level, were included in the research. In this context, the participants were informed about the voluntary participation expected through the questionnaire form and the ethical declaration requirements. Afterward, employees were invited to fill out the survey form with the support of the human resources department of the relevant bank, and a total of 390 survey forms were returned as a result of periodic reminders. After excluding 14 improper questionnaires, the remaining 376 were included in the analysis. Data were collected between February and June 2022.

The demographic characteristics of the participants are presented in Table 1. According to the findings, most participants were female (65.2%), and most were 21–30 years of age. When the participants were compared in terms of marital status, it was observed that there was almost a balanced distribution as 51.3% of the participants were single and 48.7% of the participants were married. Furthermore, finally, the education level and professional experience were taken into consideration. The majority of participants were undergraduates, and most of the participants had 1–5 years of professional experience.

**Table 1:** Demographic characteristics of respondents (*N* = 376).

Variable	Category	Frequency (f)	Percentage (%)
Gender	Male	131	34.8
	Female	245	65.2
Age	21–30 years old	176	46.8
	31–40 years old	136	36.2
	41–50 years old	64	17.0
Marital status	Single	193	51.3
	Married	183	48.7
Education level	Undergraduate	338	89.9
	Graduate	38	10.1
Professional experience	1 year or below	51	13.6
	1–5 years	119	31.6
	6–10 years	89	23.7
	11–15 years	44	11.7
	16 years or above	73	19.4

### 3.2 Measures

The existing literature was examined to adapt the appropriate constructs for measuring the study’s variables. As the scales accessed were originally in English, they were translated into Turkish using a blind translation-back-translation as described by Brislin (1976). In this context, the researchers first translated the relevant scales into Turkish. Then, the translated scales were translated back into English by experts trained in English. Finally, the final versions of the scales to be applied in the study were formed by comparing the two translations. Unless otherwise stated, the five-point Likert type of measurement with “1 = Strongly Disagree, 2 = Disagree, 3 = Neither Agree nor Disagree, 4 = Agree, to 5 = Strongly Disagree” were used. The scales used in the research are explained below (Table 2).

**Table 2:** Scales used in the study.

Scale	Developed by	Number of Items
The self-esteem scale	Rosenberg (1965)	10
The Clance impostor phenomenon scale	Clance (1985)	20
Career satisfaction scale	Greenhaus et al. (1990)	5
Proactive personality scale	Seibert et al. (2001)	10

#### 3.2.1 Self-esteem

A 10-item scale developed by Rosenberg (1965) was used in this study to measure self-esteem. This scale was preferred as it is the most cited and used scale in studies on self-esteem (Blascovich and Tomaka, 1991). Respondents were required to rate the statements by how strongly they agreed with each statement. The sample scale items are “On the whole, I am satisfied with myself,” “I am able to do things as well as most other people,” and “I take a positive attitude toward myself.” The scale has been measured as a single factor. It was found that the self-esteem scale had a good construct validity (*χ*^2^/df = 2.591, RMSEA = 0.065, CFI = 0.965, and IFI = 0.965) as a result of confirmatory factor analysis.

#### 3.2.2 Impostor phenomenon

The Clance Impostor Phenomenon Scale (CIPS), developed by Clance (1985) and validated by Chrisman (1994), was used to measure the participants’ impostor phenomenon. The scale has 20-item. Before using the CIPS scale, Dr. Clance was contacted and permission was obtained to use the scale in this study. Respondents were required to rate the statements by how strongly they agreed with each statement. The sample scale items are “I can give the impression that I’m more competent than I really am” and “At times, I feel my success has been due to some kind of luck.” As proposed by previous research (French et al., 2008), CIPS was measured as a single factor over the total score. It was seen that CIPS had a good construct validity (*χ*^2^/df = 2.702, RMSEA = 0.067, CFI = 0.913, and IFI = 0.914) as a result of confirmatory factor analysis.

#### 3.2.3 Career satisfaction

Career satisfaction was measured using a 5-item scale developed by Greenhaus et al. (1990). Respondents were required to rate the statements by how strongly they agreed with each statement. The sample scale items are “I am satisfied with the success that I have achieved in my career,” “I am satisfied with the progress that I have made toward meeting my goals for achievement.” The scale has been measured as a single factor. It was found that the career satisfaction scale had a good construct validity (*χ*^2^/df = 1.811, RMSEA = 0.047, CFI = 0.999, and IFI = 0.999) as a result of confirmatory factor analysis.

#### 3.2.4 Proactive personality

To measure the proactive personality, Seibert et al. (2001) 10-item scale, which was the short version of Bateman and Crant’s (1993) proactive personality scale, was used. Respondents were required to rate the statements by how strongly they agreed with each statement. The sample scale items are “I am constantly on the lookout for new ways to improve my life” and “I am always looking for better ways to do things.” The scale has been measured as a single factor. It was seen that the proactive personality scale had a good construct validity (*χ*^2^/df = 2.959, RMSEA = 0.072, CFI = 0.942, and IFI = 0.943) as a result of confirmatory factor analysis.

#### 3.2.5 Control variables

In this study, gender, age, marital status, education level and professional experience were named as control variables. Gender, marital status and education level were assessed with two levels, while age was assessed with three. Professional experience was assessed with five levels.

### 3.3 Statistical analysis method

The data gathered for this study was analyzed by using SPSS, PROCESS Macro and AMOS statistical analysis programs. To calculate the internal consistency of the items, Cronbach’s alpha coefficient was used. Descriptive statistics, correlations and hierarchical regression analysis were conducted using SPSS. The statistical program AMOS was utilized to analyze the model fit statistics for measurement model assessment. Moreover, PROCESS Macro was conducted to test the hypotheses. Specifically, within the scope of the models proposed by Hayes (2017), Model 1 for moderation effect, Model 4 for mediation effect and Model 14 for moderated mediation were employed.

## 4 Results

### 4.1 Reliability and validity

Confirmatory factor analysis (CFA) was employed to examine the quality of the factor structure, also named as a measurement model that depicts the relationships between latent factors and measured variables (Yang, 2005). As the model index had a poor fit, the modifications suggested by the AMOS program were applied. There were two deleted items for the self-esteem variable, the impostor phenomenon variable had five deleted items, the career satisfaction variable had no deleted items, and the proactive personality scale had three deleted items due to the low factor loadings. The results obtained from the final four-factor model consisted of self-esteem, career satisfaction, impostor phenomenon and proactive personality, revealing that the measurement model had a good model value, as presented in Table 3.

**Table 3:** Model index summary of the research model.

Index	Model value	Threshold level		Goodness to fit level
		Good fit	Acceptable	
*χ*^2^/df	2.622	≤3	≤4–5	Good Fit
CFI	0.902	≥0.95	≥0.90	Acceptable
IFI	0.903	≥0.95	≥0.90	Acceptable
RMSEA	0.066	≤0.05	≤0.08	Acceptable

The reliability and validity values of the scale are indicated in Table 4. Construct validity was assessed using Cronbach’s alpha and Composite Reliability (CR). As Cronbach’s alpha coefficient of each scale was higher than the minimum level of 0.70 (Nunnally and Bernstein, 1994) and CR ranged from 0.80 to 0.91, above the 0.70 benchmarks (Hair et al., 2010), construct validity for each construct in the study was established. Discriminant validity was also computed using the HTMT ratio. Since all HTMT ratios were less than the required limit of 0.85 (Henseler et al., 2015), the discriminant validity of the scales in this study was established.

**Table 4:** Reliability and validity of the scales.

Scale	CR	AVE	Discriminant validity	Cronbach’s *α*
Self-esteem	0.87	0.54	0.73	0.873
Career satisfaction	0.91	0.68	0.82	0.906
Impostor phenomenon	0.90	0.55	0.74	0.907
Proactive personality	0.80	0.63	0.79	0.779

There is a consensus among researchers on behavioral issues that the common method variance, which explains the variance attributed to the measurement method, is a potential issue, especially in studies which research data is obtained from a single respondent using a single questionnaire tool (Podsakoff et al., 2003; Podsakoff and Organ, 1986). Accordingly, research results will inevitably be affected by common method variance (Xu et al., 2023). To prevent common method variance, several methods can be utilized (Baumgartner et al., 2021; Baumgartner and Weijters, 2021; Becker et al., 2009; Malhotra et al., 2006). For this study, the marker variable approach recommended for use by previous studies (Lindell and Whitney, 2001; Malhotra et al., 2006; Gorrell et al., 2011) was applied. The marker variable approach involves a special variable that is intentionally included in the study, different from the other variables subject to the research (Malhotra et al., 2006). This approach is accepted as a good option for quantifying CMV when only one method (for instance, the questionnaire technique) is used to gather data for any study (Gorrell et al., 2011). Since the findings obtained as a result of the analyses conducted using the AMOS program revealed that the marker variable had a low correlation with all variables, common method variance was not an issue in this study.

### 4.2 Descriptive statistics

The correlation values between the study variables, namely self-esteem, career satisfaction, impostor phenomenon and proactive personality, and the descriptive statistics of the related variables were exhibited in Table 5.

**Table 5:** Means, standard deviations and correlations between variables.

Variables	Mean	SD	1	2	3	4	5	6	7	8	9
1. Gender	1.60	0.49	–								
2. Age	32.34	7.27	−0.176*	–							
3. Marital status	1.51	0.50	0.075	−0.429**	–						–
4. Education	1.13	0.34	−0.052	0.289**	−0.039	–					
5. Experience	2.88	1.35	−0.107	0.882**	−0.434**	0.288**	–				
6. Self-esteem	3.88	0.61	0.141*	0.070	−0.093	0.006	0.158**	–			
7. Career satisfaction	3.50	0.81	0.068	−0.131*	0.017	0.015	−0.015	0.325**	–		
8. Impostor phenomenon	2.54	0.60	0.064	−0.088	0.139*	0.007	−0.138*	−0.660**	−0.154**	**–**	
9. Proactive personality	4.24	0.39	0.147**	−0.167**	0.045	−0.075	−0.125*	0.449**	0.380**	−0.272**	–

Note. ** *p* values are significant at 0.01.

According to the results presented in Table 5, self-esteem is significantly and positively correlated with career satisfaction (*r* = 0.325) and proactive personality (*r* = 0.449), and negatively correlated with the impostor phenomenon (*r* = −0.660). Career satisfaction is significantly and negatively correlated with the impostor phenomenon (*r* = −0.154), and positively correlated with proactive personality (*r* = 0.380). Lastly, the impostor phenomenon significantly and negatively correlated with proactive personality (*r* = −0.272). As it can be followed from Table 5, good correlation values were found between all the variables examined in the study. Based on these findings, other analyses were carried out to realize the main objective of the research.

### 4.3 Hypotheses testing

#### 4.3.1 Test of direct effects

Regression analysis was conducted to analyze hypotheses 1, hypotheses 2 and 3 of the study, and the obtained findings were summarized in Table 6.

**Table 6:** Regression analysis result.

	Model 1			Model 2			Model 3		
	Career satisfaction			Impostor phenomenon			Career satisfaction		
	*β*	SE	*t*	*β*	SE	*t*	*β*	SE	*t*
Gender	−0.079	0.084	−1.582	0.152	0.051	3,804	−0.036	0.087	−0.693
Age	−0.370	0.010	−3.948	0.022	0.006	0.291	−0.384	0.011	−3.954
Marital status	−0.025	0.089	−0.449	0.090	0.054	2,023	−0.028	0.093	−0.484
Education level	0.066	0.134	1.309	−0.021	0.082	−0.520	0.060	0.139	1.158
Professional experience	0.271	0.058	2.844	0.025	0.036	0.325	0.304	0.060	3.085
Self-esteem	0.289***	0.068	5.863	−0.650***	0.042	−16.446			
Impostor phenomenon							−0.131**	0.067	−0.036
*R^2^*	0.127			0.439			0.062		
*F*	8.930***			48.209***			4.067***		
*Result of Hypothesis*	H_1_ supported			H_2_ supported			H_3_ supported		

After controlling gender, age, marital status, education level and professional experience, the results revealed that self-esteem was significantly and positively related to career satisfaction (*β* = 0.289, *p* < 0.001). Thus, hypothesis 1 was supported. It has been found that self-esteem’s negative effect on the impostor phenomenon was significant (*β* = −0.650, *p* < 0.001). Hence, hypothesis 2 was supported. Lastly, Table 6 indicates that the impostor phenomenon is significantly and negatively related to career satisfaction (= − 0.131, *p* < 0.01). Accordingly, hypothesis 3 of the study was also supported.

#### 4.3.2 Test of mediating effect

The fourth hypothesis of the study about the relationship between self-esteem and career satisfaction will be mediated by the impostor phenomenon was analyzed by PROCESS Macro bootstrapping-based procedure. After employing Model 4 proposed by Hayes (2017), it was found that the mediating effect of the impostor phenomenon showed a confidence interval at a value of zero in between (*β* = −0.0655, Boot SE = 0.0683, 95% CI = [−0.2012, 0.0695]), which proves that hypothesis 4 was not supported (see Table 7).

**Table 7:** The results of mediating effect.

Indirect effect	*β*	%95 Boot CI		Boot SE	*t*	Sig.	Result of Hypothesis 4
		LLCI	ULCI				
SE → IP → CS	−0.0655	−0.2012	0.0695	0.0683	1.149	0.251	Not supported

SE, Self-esteem; IP, Impostor phenomenon; CS, Career satisfaction; CI, Confidence interval; LLCI, Lower limit of the confidence interval; ULCI, Upper limit of the confidence interval.

#### 4.3.3 Test of moderating effect

Regression analysis based on the bootstrap method by Model 1 of Hayes (2017) was conducted to test the moderating role of proactive personality in the effect of the impostor phenomenon on career satisfaction. In the analyses applied using PROCESS Macro developed by Hayes (2017), the bootstrap technique and 5,000 resampling options were chosen. According to the regression results presented in Table 8, it was identified that all variables included in the analysis explained approximately 15% (*R*^2^ = 0.147) of the change in career satisfaction. The interactional effect (moderating effect) of the impostor phenomenon and proactive personality on career satisfaction was significant (*β* = −0.3711, Boot SE = 0.1126, 95% CI = [−0.5925, −0.1496]). Therefore, Hypothesis 5 was supported.

**Table 8:** Moderation effect.

Path	*β*	%95 Boot CI		Boot SE	*t*	sig.	Result of Hypothesis 5
		LLCI	ULCI				
IP*PP → CS	−0.3711	−0.5925	−0.1496	0.1126	−3.2949	0.001	Supported

IP, Impostor phenomenon, PP, Proactive Personality; CS, Career satisfaction; CI, Confidence interval; LLCI, Lower limit of the confidence interval; ULCI, Upper limit of the confidence interval.

**Figure 2:**
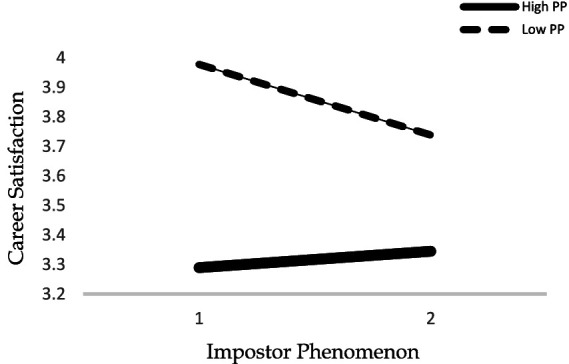
Proactive personality as a moderator between impostor phenomenon and career satisfaction relationship.

The interaction between variables was graphically displayed in Figure 2 below. The graphic can be interpreted as the effect of the impostor phenomenon on career satisfaction becoming weaker when the proactive personality was high. In other words, the career satisfaction of the employees who experience the impostor phenomenon decreases, but having a proactive personality changes this association. Career satisfaction increases due to the proactive personality even if the employee experiences the impostor phenomenon.

#### 4.3.4 Test of moderated mediation effect

The moderated mediation effect proposed in the research model of this study was analyzed with Model 14 of Hayes (2017). This model suggests that the indirect effect of self-esteem on career satisfaction via the impostor phenomenon is conditional on proactive personality. The condition of moderated mediation is proved when the conditional indirect effect of self-esteem on career satisfaction via the impostor phenomenon differs in levels of proactive personality. To examine the moderated mediation hypothesis, the index of the moderated mediation observed as exhibited in Table 9, and as this index excluded the value of zero (Conditional indirect effect = 0.2766, Boot SE = 0.0901, 95% CI = [0.1057, 0.4580]), it was found out that the result was statistically significant. Accordingly, Hypothesis 6 was supported.

**Table 9:** Index of moderated mediation.

Moderator = PP	Index	Boot SE	%95 Boot CI	
			LLCI	ULCI
SE → IP → CS	0.2766	0.0901	0.1057	0.4580

PP, Proactive personality; SE, Self-esteem; IP, Impostor phenomenon; CS, Career satisfaction; CI, Confidence interval; LL, Lower limit; UL, Upper limit.

The findings in Table 10 below demonstrated that the indirect effect of self-esteem on career satisfaction via impostor phenomenon was significant when proactive personality is low (Conditional indirect effect = −0.1511, Boot SE = 0.0591, 95% CI = [−0.2636, −0.0304]) but was not significant when proactive personality is high (Conditional indirect effect = 0.0426, Boot SE = 0.0835, 95% CI = [−0.1037, 0.2245]).

**Table 10:** Moderated mediation effect.

Moderator = PP	Indirect effect	Boot SE	%95 Boot CI	
			LLCI	ULCI
−1 SD	−0.1511	0.0591	−0.2636	−0.0304
0	−0.0957	0.0606	−0.2096	0.0267
+1 SD	0.0426	0.0835	−0.1037	0.2245

Independent variable: Self-esteem; Dependent variable: Career satisfaction; Mediator: Impostor phenomenon; Moderator: Proactive personality; CI: Confidence interval; LL: Lower limit; UL: Upper limit.

The findings in Tables 9, 10 indicate that the mediating effect of the impostor phenomenon on the relationship between self-esteem and career satisfaction is moderated by proactive personality. That is, the effect of self-esteem on career satisfaction via the impostor phenomenon is only significant for employees with low levels of proactive personality. According to this result, the mediating effect of the impostor phenomenon in the effect of self-esteem on career satisfaction is negatively related when proactive personality trait is low. When individuals with low proactive personality exhibit high impostor phenomenon, this emotion affects their career satisfaction levels more negatively than individuals with high proactive personality.

## 5 Discussion

This research was conducted to investigate the mediating effect of the impostor phenomenon in the relationship between self-esteem and career satisfaction, along with suggesting that the proactive personality moderated the indirect effect of self-esteem on career satisfaction through the impostor phenomenon. The results obtained from the study were interpreted as follows.

First, the direct effects of self-esteem, career satisfaction and impostor phenomenon were analyzed within the main three hypotheses. Hypothesis 1, indicating that self-esteem is positively associated with career satisfaction, was verified. This result supported the studies previously conducted in this field (Sabelnikova, 2021; Cai et al., 2015; Mocheche et al., 2017; Zafar et al., 2014; Kuster et al., 2013; Ahmed, 2012; Kammeyer-Mueller et al., 2008; Alavi and Askaripur, 2003; Garske, 1996; Westaway et al., 1996). The conclusion drawn from this result is that employees with high self-esteem tend to be satisfied with their careers. Hypothesis 2, stating that self-esteem is negatively associated with the impostor phenomenon, was supported. This result is consistent with previous research (Mascarenhas et al., 2019; Lige et al., 2017; Schubert and Bowker, 2019; Neureiter and Traut-Mattausch, 2016; Sonnak and Towell, 2001; Topping and Kimmel, 1985) within the available literature. This finding provides supportive evidence that employees with high self-esteem would not feel the impostor phenomenon since they had self-confidence and awareness of their capabilities and competencies. Hypothesis 3, indicating that the impostor phenomenon is negatively associated with career satisfaction, was verified. This finding was consistent with previous studies conducted in this field (Sharma, 2018; Neureiter and Traut-Mattausch, 2016). The conclusion drawn from this result is that employees with the impostor phenomenon would try to be a way of being noticed by others, so they could not be satisfied with their careers as they thought they would not deserve such a career. Second, the indirect impostor phenomenon in the relationship between self-esteem and career satisfaction was analyzed. Within this context, Hypothesis 4, emphasizing that the impostor phenomenon plays a mediating role in the relationship between self-esteem and career satisfaction, was not supported. According to this result, there is no mediating effect of the impostor phenomenon on the effect of self-esteem on career satisfaction. This result is unexpected in terms of research design. It is an expected result that as an individual’s self-esteem level decreases, the impostor phenomenon increases, and this situation negatively affects career satisfaction. However, different variables may affect the career satisfaction of the sample participating in the research may have affected this result. However, other variables may influence this relationship, as the indirect effect of the impostor phenomenon in the relationship between self-esteem and career satisfaction is not significant. Hypothesis 5, highlighting that proactive personality moderates the relationship between the impostor phenomenon and career satisfaction, was supported. The conclusion that can be drawn from this result is that a decline in the career satisfaction of the employees feeling impostor phenomenon may increase when they have highly proactive personalities. Hypothesis 6, indicating that proactive personality moderated the mediation role of the impostor phenomenon in the relationship between self-esteem and career satisfaction, was supported. According to this result, the mediating effect of the impostor phenomenon in the effect of self-esteem on career satisfaction is negatively related when proactive personality trait is low. Individuals with low proactive personality have difficulty taking action and have trouble taking initiative. When these individuals exhibit high impostor phenomenon, this emotion may affect their career satisfaction levels more negatively than individuals with high proactive personality. Even if individuals with high proactive personality experience the impostor phenomenon, they are likely to be more successful in their business life and thus feel higher levels of career satisfaction as they have higher ability to struggle due to their individual characteristics such as taking action, taking initiative and influencing the environment. Both impostor phenomenon and proactive personality affecting the behaviors and attitudes of individuals in business life, have an impact on possible negative emotions and feelings of deficiency.

### 5.1 Theoretical and practical implications

This study has some implications both for literature and organizations. Theoretical and practical implications are explained below.

#### 5.1.1 Theoretical implications

This study has several theoretical implications for the available literature. The first contribution of the study lies in the roots of addressing an under-researched topic within the associations of self-esteem, impostor phenomenon, career satisfaction and proactive personality together. The results of the study broaden the research field related to study’ variables. The research variables, being understudied topics, have opened a novel path for studies to be conducted in different occupational fields with a similar research design. Enriching the available literature is expected to widen the scope of the research field, especially the impostor phenomenon. The other theoretical contribution is that this study will help the conceptualization of impostor phenomenon and its associations with its antecedences and consequences. Understanding the reflections of the impostor phenomenon in professional business life beyond an individual syndrome and being able to associate its results with different variables will make significant contributions at the organizational level.

The study’s third considerable contribution is that its findings strengthen Self-Determination Theory (SDT) and Social Identity Theory (SIT). SDT suggests autonomous and competent individuals are more likely to experience higher career satisfaction. When individuals feel in control of their work and have the necessary skills to achieve their goals, they are more likely to experience a sense of fulfillment and satisfaction from their work. Consistent with our H1 hypothesis, individuals with high self-esteem are more likely to pursue career paths that align with their interests and values, resulting in greater career satisfaction. However, according to SDT, individuals who feel autonomous and competent in their work are less likely to experience the impostor phenomenon. When individuals feel in control of their work and have the necessary skills to achieve their goals, they are less likely to experience self-doubt and feelings of inadequacy. Individuals with low self-esteem are more likely to experience the impostor phenomenon in this context. They may doubt their abilities and attribute their successes to external factors such as luck or help from others. In contrast, individuals with high self-esteem are more likely to attribute their successes to their abilities and efforts, leading to greater self-confidence and reduced susceptibility to the impostor phenomenon. Our H3 hypothesis reveals that impostor phenomenon can undermine an individual’s basic psychological needs, decreasing career satisfaction. In accordance with SDT, when an individual experiences the impostor phenomenon, their sense of competence and autonomy can be undermined, leading to decreased motivation and engagement in their work. On the other hand, SIT suggests that an individual’s membership in social groups can influence their self-esteem and career satisfaction. Individuals who belong to social groups highly regarded by society may experience higher self-esteem and career satisfaction levels due to the social status associated with their occupation. In contrast, individuals who belong to social groups that are stigmatized or marginalized may experience lower levels of self-esteem and career satisfaction due to the discrimination and bias they may face. SIT also provides a useful framework for understanding the relationship between self-esteem and the impostor phenomenon. According to SIT, when an individual’s social identity is threatened or undermined, it can decrease their self-esteem and increase their susceptibility to the impostor phenomenon. Because individuals with high self-esteem generally have a positive self-image and are more likely to feel confident and competent in their abilities. In contrast, individuals with low self-esteem may doubt their abilities and feel inadequacy in their personal and professional lives.

#### 5.1.2 Practical implications

The implications of this study for practice can be explained as follows. First of all, human resources departments of organizations can review their recruitment processes and focus on their procedures for recruitment and selection of employees. In this context, more comprehensive and detailed tests can be used to select employees with high self-esteem and proactive personality, policies and procedures can be followed, and managers can be encouraged to set more selective criteria. In particular, organizations that want to ensure employees’ career satisfaction should focus on recruiting employees with high self-esteem and proactive personality. Second, increasing pressures and stress in today’s rapidly changing and intensely competitive business life may cause employees to experience the impostor phenomenon. In addition, situations such as feelings of inadequacy and loss of competence may result in employees’ intrinsic acceptance and internalization of the impostor phenomenon. In this context, organizations that need success-oriented and highly motivated employees to achieve their goals should plan training programs that will raise awareness about the impostor phenomenon, and reveal effective ways to cope with this syndrome. Through this awareness, it can be ensured that employees comprehend this issue and have a perception of reality. In addition, by ensuring rotation between different departments, improvement areas can be created where employees can test their performances in various fields. In this way, employees can improve themselves, increase their awareness and reduce their tendency to see deficiencies in themselves by understanding the differences. As a result, employees can cope with this syndrome by increasing their knowledge about the impostor phenomenon. Third, even if employees experience the impostor phenomenon, their high self-esteem and proactive personality make them more likely to experience career satisfaction. From the perspective of today’s business world, managers have a great responsibility to sustain this reality. Managers should not only be goal and performance-orientated, but they should also have aspects that consider employees’ welfare, strive to make them feel good and act consciously in this regard. It is of great importance that managers are far from the impostor phenomenon, internalize success and aim to spread wellbeing throughout the organization. Finally, by creating a team spirit within the organization, it can be ensured that managers provide individual support to employees on the impostor phenomenon by creating a climate of interaction and information sharing among employees. In this way, it may be possible for employees with high self-esteem and proactive personality, who are not affected by the impostor phenomenon syndrome and who experience career satisfaction. As a result, it supports the realization of the predetermined goals of the organization.

### 5.2 Limitations and future directions

Although this study examined a topic that has yet to be emphasized to date, it has acknowledged the existence of several limitations in this research, which can also lead to future research directions.

First, since the research data were collected with the survey technique from quantitative methods, a single method was utilized. Evaluating the research variables through the findings obtained within the scope of qualitative methods may reveal different results. Therefore, mixed methods using both quantitative and qualitative methods may be preferred in future research. In addition, this research was conducted as a cross-sectional study. In this respect, future research may include longitudinal studies to reveal the connection between the relevant variables. The second limitation is about the scales used in the study. The CIPS scale used in this study was measured and interpreted without taking into account the sub-dimensions used to reveal the existence of the impostor phenomenon, as preferred in different studies (French et al., 2008; Neufeld et al., 2022; Mak et al., 2019). In order to examine the relationship between the impostor phenomenon and different variables in more depth, future research may focus on the measurement and interpretation of the CIPS scale within the scope of its sub-dimensions. Third, the fact that the research data were collected from the employees working in a single bank operating in the banking sector constitutes a limitation for the generalizability of the research results. In addition, the fact that the data were gathered only from the banking sector and especially from a private bank supports the existence of this limitation. Therefore, it may be suggested that future research should be conducted in different sectors, and especially public bank employees should be analyzed. The fourth limitation of the study was that ignoring the participants’ positions. Choosing employees with non-managerial positions might cause a limitation. Therefore, different results may have been reported if the sample for this survey had been constituted of managers at higher levels of the organizations. Hence, in future research, it is recommended that organizations of different scales should be preferred, as well as managers working in top management should be selected as a sample. The fifth limitation is that the study is conducted in the Turkish context, where cultural differences should be considered as a decisive attitude. Future research could therefore include extending this work to other cultures. Finally, other researchers may conduct further studies on investigating the associations between the impostor phenomenon and the other antecedent and consequent variables such as perfectionism, turnover intention, self-control, perceived over-qualification, self-compassion, etc. Especially in future studies, our model can be tested again by considering self-efficacy as an independent variable or career commitment as a dependent variable.

## 6 Conclusion

This study intended to test a moderated mediation model of self-esteem, impostor phenomenon and proactive personality for career satisfaction. The conclusions that can be drawn from this study are as follows: First, within the direct effect, it was found out that self-esteem predicted career satisfaction positively and impostor phenomenon negatively; and impostor phenomenon negatively predicted career satisfaction; and second, it was revealed that impostor phenomenon did not play a mediating role in the association between self-esteem and career satisfaction; and third, proactive personality assigned as a moderator on the association between impostor phenomenon and career satisfaction; and fourth, support was found for the moderated mediation model tested in this study. The conclusions suggest that employees with high self-esteem were more satisfied with their careers. Employees with low self-esteem felt the impostor phenomenon more often than employees with high self-esteem. Highly proactive employees were more satisfied with their careers even if they felt the impostor phenomenon. Organizations need to create and follow the procedures that might help them to recruit employees with high self-esteem and proactive personality.

Building on these findings, we can underscore the importance of fostering a work environment that actively promotes the development of self-esteem and proactive personalities. These characteristics, as shown by the study, are crucial for higher career satisfaction and can serve as antidotes to the impostor phenomenon. Furthermore, leadership strategies should consider these psychological constructs when designing staff training and development programs. On a broader scale, this research signifies the complexity of the psychological factors at play in career satisfaction. The interplay between self-esteem, the impostor phenomenon, and proactive personality, revealed in this study, provides valuable insights into career satisfaction’s dynamics. Further studies may wish to explore these relationships in different cultural and professional contexts, adding to the richness and applicability of these findings in diverse environments.

## Data Availability

The original contributions presented in the study are included in the article/supplementary material, further inquiries can be directed to the corresponding author.
